# Suicidal risk associated with finasteride versus dutasteride among men treated for benign prostatic hyperplasia: nationwide cohort study

**DOI:** 10.1038/s41598-023-32356-3

**Published:** 2023-03-31

**Authors:** Moussa Laanani, Alain Weill, Fabrice Jollant, Mahmoud Zureik, Rosemary Dray-Spira

**Affiliations:** 1 Epiphare (French National Medicines Agency ANSM and French National Health Insurance CNAM), Saint-Denis, France; 2 Present Address: French National Health Insurance (CNAM), Paris, France; 3 Université Paris-Saclay & CHU Bicêtre, AP-HP, Le Kremlin-Bicêtre, France; 4 Nîmes University Hospital (CHU), Nîmes, France; 5 Department of psychiatry, McGill Group for Suicide Studies, McGill University, Montréal, Canada; 6 Moods Team, INSERM UMR-1018, CESP, Le Kremlin-Bicêtre, France

**Keywords:** Epidemiology, Public health, Prostate, Psychiatric disorders

## Abstract

Finasteride, a 5α-reductase inhibitor used in benign prostatic hyperplasia and androgenetic alopecia, has been associated with an increased suicidal risk, whereas it is unclear whether such risk is similar to that for another 5α-reductase inhibitor, dutasteride. We aimed to assess the risk of suicidal behaviours with finasteride relative to dutasteride. A nationwide cohort study was conducted using the French National Health Data System (SNDS). Men aged 50 years or older initiating finasteride 5 mg or dutasteride 0.5 mg in France between 01-01-2012 and 30-06-2016 were included and followed until outcome (suicide death identified from death certificate or self-harm hospitalisation), treatment discontinuation or switch, death, or 31-12-2016. Self-harm by violent means or resulting in admission to an intensive care unit were also examined. Cox proportional hazards models controlled for age and psychiatric and non-psychiatric conditions by inverse probability of treatment weighting (IPTW). Analyses were stratified according to psychiatric history. The study compared 69,786 finasteride new users to 217,577 dutasteride new users (median age: 72.0 years [Q1–Q3 = 64.5–80.2] vs. 71.1 [Q1–Q3 = 65.0–79.2]). During follow-up, 18 suicide deaths (0.57/1000 person-years) and 34 self-harm hospitalisations (1.08/1000) occurred among finasteride users versus 47 deaths (0.43/1000) and 87 hospitalisations (0.79/1000) among dutasteride users. Overall, finasteride was not associated with an increased risk of any suicidal outcome (IPTW-adjusted Hazard Ratio = 1.21 [95% Confidence Interval  .87–1.67]), suicide death or self-harm hospitalisation. However, among individuals with a history of mood disorders, finasteride was associated with an increased risk of any suicidal outcome (25 versus 46 events; HR = 1.64 [95% CI 1.00–2.68]), suicide death (8 versus 10 events; HR = 2.71 [95% CI 1.07–6.91]), self-harm by violent means (6 versus 6 events; HR = 3.11 [95% CI 1.01–9.61]), and self-harm with admission to an intensive care unit (7 versus 5 events; HR = 3.97 [95% CI 1.26–12.5]). None of these risks was significantly increased among individuals without a psychiatric history. These findings do not support an increased risk of suicide with finasteride used in the treatment of benign prostatic hyperplasia. However, an increased risk cannot be excluded among men with a history of mood disorder, but this result based on a limited number of events should be interpreted with caution.

## Introduction

Finasteride is a 5α-reductase inhibitor indicated for men for the treatment of benign prostatic hyperplasia (BPH) at 5 mg/day and for androgenetic alopecia at 1 mg/day. Psychiatric adverse events, notably low mood and suicidal ideation, have been observed, leading to the following mention in the European summaries of product characteristics: “Mood alterations including depressed mood, depression and, less frequently, suicidal ideation have been reported in patients treated with finasteride 5 [or 1] mg”. On the US label, only depression is mentioned, not suicidal ideation^1^. The psychiatric and suicidal adverse effects of finasteride did not appear during clinical trials but were suggested and then investigated with post-marketing observational studies^2–9^. Although they could be attributable to the sexual side effects of the drug (highlighted before marketing)^10–12^, there is some evidence to suggest the involvement of distinct pathophysiological pathways for sexual and psychiatric complications. Observational studies have described cases of patients exposed to finasteride developing depressive disorders without sexual disorders^13,14^. Biological studies have shown that finasteride crosses the blood–brain barrier and can be involved in mood changes by affecting the concentration of neuro-steroids and their metabolites in the cerebrospinal fluid^15–18^. Psychiatric and suicidal events can appear early after treatment initiation^13,14^ and persist long after treatment discontinuation, as described in the “post-finasteride syndrome”, consisting of an association of sexual, physical, and psychological symptoms that develop during or after finasteride exposure and persist after discontinuation^7,19^. Regarding psychological symptoms, clinically significant depression was reported in 50% of a sample of men with post-finasteride syndrome, and anxiety in 34%^20^.

However, the interpretation of findings from observational studies is debated. The major point of discussion concerns the methodological quality of certain studies on the suicidal risk of finasteride, and, in particular, the selection bias than can be involved when recruiting participants from propeciahelp.com, a website dedicated to post-finasteride syndrome, or the Post-Finasteride Foundation^2,3,7^. Although large observational studies have reported reassuring results about the depressive risk of finasteride^21–23^, less reassuring results emerged from a study by Welk et al. on the suicidal risk of 5α-reductase inhibitors^6^, of which the methodological quality has been underlined by a number of authors^24–26^. Comparing 93,000 men with BPH exposed to 5α-reductase inhibitors to 93,000 matched unexposed men, they highlighted an increased risk of self-harm, but not suicide death, during the first 18 months after treatment initiation. On another note, a recent Korean population-based study did not find an increased risk of suicide associated with long-term treatment, but did not assess the short-term effects of exposure to finasteride^9^.

Dutasteride is another 5α-reductase inhibitor indicated for the treatment of BPH (at 0.5 mg/day), usually as a second-line therapy after α-blockers, similarly to finasteride^27^. Its elimination half-life is much longer than that of finasteride (5 weeks vs. 5 to 8 h). It also binds more highly to plasma proteins (99% vs. 90%)^28^. It is commonly accepted that finasteride and dutasteride show similar efficacy and sexual safety profiles^29^ but suicidal risk is not mentioned in the European summaries of the product characteristics of dutasteride. Studies assessing the suicidal risk of dutasteride are scare, and do not suggest an increased risk^5,30,31^. Comparing the risk of suicidal events in finasteride users with dutasteride users in the treatment of BPH has several advantages. Although BPH affects the quality of life and may induce depressive symptoms^32^, the confounding that the indication of treatment may induce may be lower than when assessing treatment-seeking patients with androgenetic alopecia. Thus, in terms of confounding by indication (a bias that occurs when the clinical indication for receiving the study treatment is a risk factor for the outcome of interest^33^), it may be easier to study the suicidal risk of finasteride in men with BPH than in men with androgenetic alopecia, as treatment is indicated because of the psychological impact of androgenetic alopecia (whereas it is the somatic impact of BPH that mainly drives the prescription in the first situation). In addition, finasteride and dutasteride are both in the same pharmacological class (5α-reductase inhibitors) and are indicated in the same line of treatment, thus further mitigating the risk of confounding by severity in this assessment^34^. In their study, Welk et al. investigated whether there was a discrepancy in suicidal risk between dutasteride and finasteride and did not observe any significant difference. However, their analyses did not take into account the possible switches that could occur during treatment with 5α-reductase inhibitors and only considered the drug initially prescribed during the exposure to 5α-reductase inhibitors, allowing subjects to switch between finasteride and dutasteride during follow-up. Thus, when a suicidal event occurred after a switch, it was attributed to the first drug received although it could also be attributed to the second one, leading to possible misclassification^6^.

The aim of our study was to compare the risk of suicidal events (suicide death and self-harm hospitalisation) associated with finasteride relative to that associated with dutasteride during the treatment of BPH. We used data from large and comprehensive national healthcare databases. This was all the more necessary, as suicide is a rare event, therefore necessitating the use of large representative cohorts.

## Methods

### Data sources

This population-based nationwide cohort study was based on the French National Health Data System (SNDS, for *Système National des Données de Santé*), which consists of the linkage of individual data from several databases: the National Health Insurance claims information system (SNIIRAM), the national hospital discharge database (PMSI), and the national causes-of-death registry. These databases provide detailed information on health insurance claims for inpatient (PMSI) and outpatient (SNIIRAM) care for 99% of the population living in France (approximately 67,000,000 people)^35^. The SNIIRAM database contains data about all outpatient services reimbursed by the National Health Insurance, including drugs (coded according to the Anatomical Therapeutic Chemical classification system, ATC), but does not provide any information about the medical indication. Patients with chronic diseases (LTD: long-term diseases), such as mood disorders, are 100% reimbursed for their health expenditures, and the diagnosis is recorded in the SNIIRAM database. Hospitalisation diagnoses that have an impact on medical care are coded in the PMSI database according to the International Classification of Diseases, 10th revision (ICD-10). It also contains information on procedures performed during hospitalisation, coded according to the French medical classification for clinical procedures (CCAM)^36^. Medical causes of death are available in the national registry for all deaths that occur in France^37^. Diagnoses (LTDs, hospitalisation diagnoses, and causes of death) are all recorded using ICD-10 codes^38^.

The SNIIRAM and PMSI databases are linked by means of a unique anonymous number allocated to each individual. Linkage of the causes-of-death registry to SNIIRAM and PMSI is indirect and deterministic, using several common variables: full date of death, month and year of birth, gender, and place of residence. The SNDS is a useful and reliable source for the assessment of drug safety ^39–44^. Further description of this database and its use is available in Tuppin et al. ^35^. This observational study based on the French healthcare databases was approved by the French Data Protection Agency (*Commission Nationale de l'Informatique et des Libertés*, regulatory decision CNIL-2016–316) and did not require patient consent or ethics committee approval. All methods were performed in accordance with the relevant guidelines and regulations.

### Participants and follow-up

The study population consisted of male subjects aged 50 years or older and treated in France with a 5α-reductase inhibitor: finasteride 5 mg (ATC code G04CB01) or dutasteride 0.5 mg (ATC codes G04CB02 and G04CA52), either alone or in combination with an alpha-blocker. Men aged 49 years or younger were not considered to limit the inclusion of individuals exposed to reimbursed 5 mg tablets of finasteride for the treatment of androgenetic alopecia instead of BPH, as 1 mg tablets are not reimbursed by the French National Health Insurance. Treatment initiation was considered between 1 January 2012 and 30 June 2016. The year 2011 was used to check for the absence of finasteride or dutasteride delivery and exclude prevalent users. Individuals were followed from treatment initiation until the event of interest, the end or switching of treatment exposure, death, or 31 December 2016, whichever occurred first, allowing at least 6 months of follow-up for every subject. The number of tablets in the drug boxes delivered was used to define the duration of drug exposure from the date of drug delivery. A 15-day slack period was allowed after the exposure period deduced from the number of tablets in a delivered drug box to account for variability in drug delivery dates. If the same drug was delivered during this slack period, exposure was considered to not be discontinued. During hospital stays, for which data on drug use is not available in the SNDS, changes in treatment (switch, discontinuation) were assumed to have occurred in the middle of hospitalisation.

The study population was restricted to National Health Insurance general scheme beneficiaries, as exhaustive information on the date of death, necessary for linkage to causes-of-death data, is only available in this insurance scheme. The general scheme is the largest health insurance scheme in France (covering 76% of the people living in France)^35^. The linkage rate to causes-of-death data is 94% in this population^45^. This scheme covers employed workers and retired, unemployed, and disadvantaged individuals. The follow-up of beneficiaries is stable, as people rarely change the insurance scheme. Other schemes are more specific and mainly cover farmers, self-employed workers, military personnel, and students^35^.

### Outcomes

Suicide deaths were identified from the national causes-of-death register (underlying cause of death ICD-10 codes X60 to X84, see Online Appendix 1). Hospitalisations (in “medicine, surgery or obstetrics” wards) for self-harm were identified from the PMSI database with the same discharge diagnostic codes. The primary outcome was a composite variable consisting of the first occurrence of either suicide death or hospitalisation for self-harm. The secondary outcomes were: (1) suicide death or (2) the first hospitalisation for self-harm during the follow-up period. Severe self-harming acts were further investigated using two definitions: (i) use of a violent means (ICD-10 codes X66-X83, including, for example, self-harm by hanging, firearm discharge, or jumping from a high place or before a moving object; see Online Appendix 1), and (ii) admission to an intensive care unit.

### Covariates

History of hospitalisation for self-harm was identified in the 3 years prior to treatment initiation. Psychiatric history was identified for depression and mood disorders, anxiety disorders, and other psychiatric diagnoses and psychotropic treatments in the year prior to treatment initiation. Identification was made using hospitalisation (“medicine, surgery or obstetrics” wards, psychiatry, or rehabilitation) or LTD diagnostic codes. Pre-existing psychiatric conditions were also be identified by psychotropic drug deliveries at three different dates during the year prior to treatment initiation. The codes used for the identification of pre-existing psychiatric conditions are presented in Online Appendix 2.

Prostatic history was identified for transurethral resection of the prostate, the use of alpha-blockers, and prostate cancer. Transurethral resection of the prostate was identified through CCAM procedure codes for hospitalisations in “medicine, surgery, or obstetrics” wards in the year prior to treatment initiation. The use of alpha-blockers was identified by at least one drug delivery in the year prior to treatment initiation. Prostate cancer was identified using hospitalisation or LTD diagnostic codes in the 3 years prior to treatment initiation. The codes used for the identification of pre-existing prostatic conditions are presented in Online Appendix 3.

Finally, the 14 conditions used in the Charlson index^46^ were identified in the SNDS database during the 3 years preceding treatment initiation, as described elsewhere^47^.

### Statistical analysis

Incidence rates per 1000 person-years were estimated for each outcome (composite outcome, suicide death, self-harm hospitalisation, self-harm by violent means, and self-harm with admission to an intensive care unit) by dividing the number of outcomes occurring during follow-up by the number of person-years of follow-up from treatment initiation to the event or censoring. Kaplan–Meier curves were plotted to describe the timing of composite outcomes, suicide deaths, and self-harm hospitalisation during the entire follow-up period and during the first 3 months.

Associations between treatment exposure and outcomes were estimated using Cox proportional hazards models. Results are presented as hazard ratios (HR) with their 95% confidence interval [95%CI]; *p*-values < 0.05 were considered statistically significant. For adjusted estimations, control for covariates measured at baseline was performed by inverse probability of treatment weighting (IPTW)^48^, given the low number of outcomes and the high number of potential measured confounders. Each individual was attributed a propensity score PS, estimated using a logistic regression model, assigning, for each individual, the conditional probability of being treated with finasteride, given the included covariates. A pseudo-population was created by weighting each individual by the inverse of the probability of receiving the treatment he actually received (i.e., 1/PS for individuals treated with finasteride, 1/(1-PS) for those treated with dutasteride). Stabilized weights, calculated by multiplying the original weights by the unadjusted probability of treatment, were used to ensure an accurate estimation of variance. Conservative 95% confidence intervals were constructed using the robust variance estimator^48^.

As suicide risk is strongest among individuals with psychiatric disorders^49,50^, analyses were performed for each considered outcome on the whole study population and on the following restricted populations: subjects without a history of a psychiatric disorder or self-harm hospitalisation, subjects with a history of a psychiatric disorder or self-harm hospitalisation, subjects with a history of self-harm hospitalisation within 3 years, and subjects with a history of mood disorders, anxiety disorders, or other psychiatric disorders. Analyses were also performed censoring the follow-up at 90 days to assess any potential trigger effect of treatment initiation on the whole study population and on patients with a history of mood disorders. Stabilized weights were re-estimated for each sub-group analysis. Sensitivity analyses were performed by further adjusting the IPTW adjusted outcome models for age as a time-varying covariate, included as a quadratic polynomial.

Statistical analyses were performed using R (V3.5.2)^51^.

### Ethics approval and consent to participate

This observational study based on the French healthcare databases was approved by the French Data Protection Agency (*Commission Nationale de l'Informatique et des Libertés*, regulatory decision CNIL-2016–316) and did not require patient consent or ethics committee approval. Patients and/or public were not involved. All methods were performed in accordance with the relevant guidelines and regulations.

## Results

**Figure 1:**
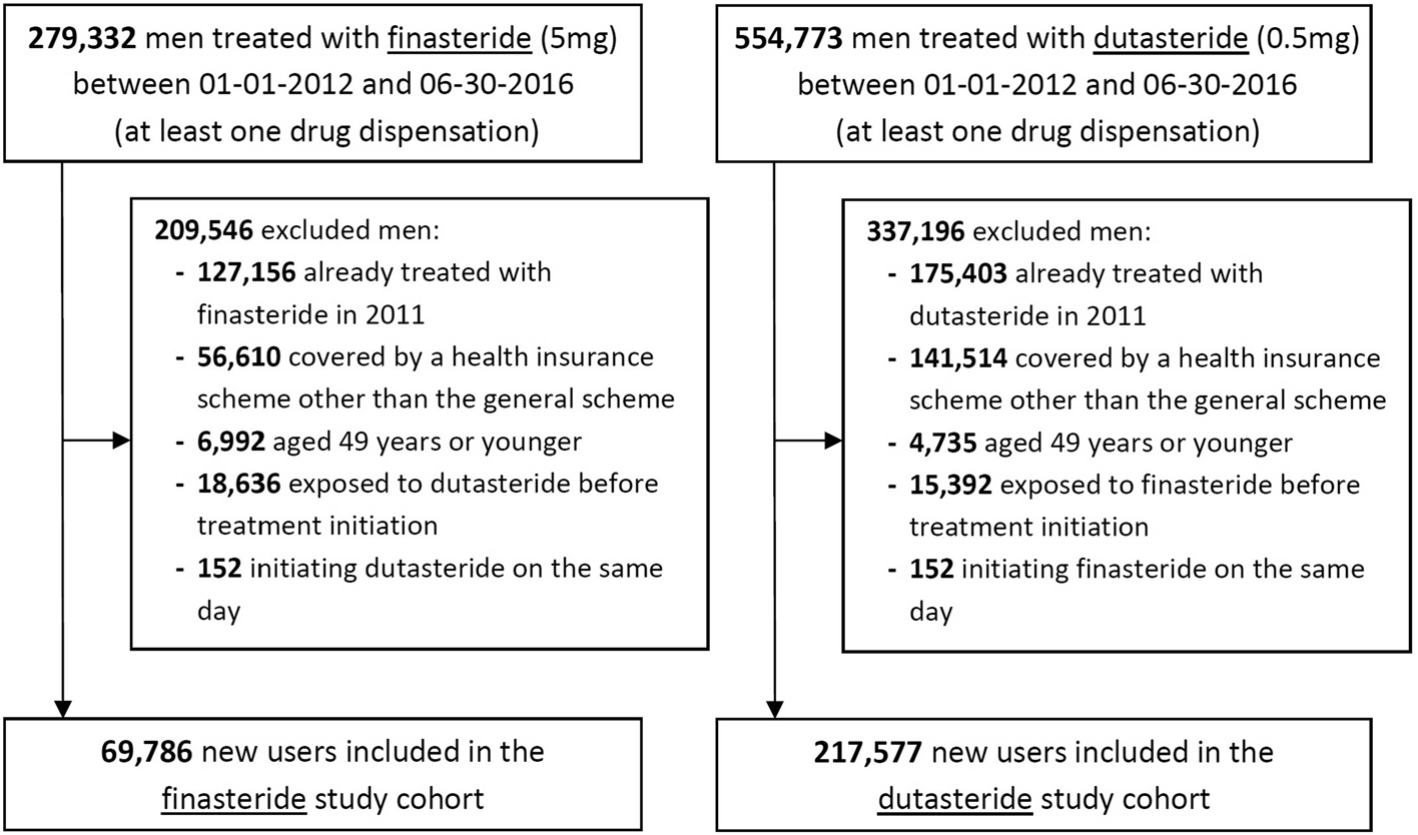
Study flow diagram.

Between 1 January 2012 and 30 June 2016, 279,332 men exposed to finasteride 5 mg and 554,773 exposed to dutasteride 0.5 mg were identified. After exclusion (mainly of men exposed to the studied drugs in 2011, or not covered by the general health insurance scheme), the final cohorts included 69,786 finasteride new users (corresponding to 31,344.9 person-years), and 217,577 dutasteride new users (110,329.4 person-years, Fig. 1).

The baseline characteristics of the two groups are presented in Table 1. The median age was slightly higher in the finasteride than dutasteride group (72.0 years vs. 71.1 years). Although pre-existing prostatic conditions and treatments were less frequent in the finasteride group, pre-existing psychiatric conditions and treatments were more frequent. Charlson index comorbidities were also generally more frequent in the finasteride group. The duration of treatment was shorter in the finasteride group.

**Table 1:** Baseline characteristics of individuals included in the groups treated with finasteride or dutasteride.

Characteristic, No. (%)	Finasteride (N = 69,786)	Dutasteride (N = 217,577)
Age (in years)		
Median [Q1-Q3]	72.0 [64.5–80.2]	71.7 [65.0–79.2]
Range	50.0–105.5	50.0–105.4
Year of treatment initiation		
2012	18,389 (26.4%)	48,788 (22.4%)
2013	15,522 (22.2%)	49,824 (22.9%)
2014	14,331 (20.5%)	48,880 (22.5%)
2015	13,936 (20.0%)	46,142 (21.2%)
2016 (January to June)	7,608 (10.9%)	23,943 (11.0%)
Pre-existing psychiatric conditions and treatments		
Any psychiatric history, including self-harm hospitalisation	19,824 (28.4%)	55,584 (25.6%)
Self-harm hospitalisation	185 (0.27%)	466 (0.21%)
Mood disorder or treatment for mood disorder	8,638 (12.4%)	23,503 (10.8%)
Anxiety disorder or treatment for anxiety disorder	10,371 (14.9%)	29,099 (13.4%)
Other psychiatric diagnoses or psychotropic treatments	10,413 (14.9%)	27,989 (12.9%)
Pre-existing prostatic conditions and treatments		
Alpha-blockers	33,885 (48.6%)	129,386 (59.5%)
Transurethral resection of the prostate	598 (0.9%)	2,054 (0.9%)
Prostate cancer	953 (1.4%)	4,922 (2.3%)
Pre-existing conditions used in the Charlson index		
Myocardial infarct	9,314 (13.4%)	27,750 (12.8%)
Congestive heart failure	5,115 (7.3%)	11,975 (5.5%)
Peripheral vascular disease	7,445 (10.7%)	21,499 (9.9%)
Cerebrovascular disease	5,515 (7.9%)	14,480 (6.7%)
Dementia	3,333 (4.8%)	6,482 (3.0%)
Chronic pulmonary disease	14,967 (21.5%)	45,075 (20.7%)
Connective tissue disease	842 (1.2%)	2,433 (1.1%)
Ulcer disease	727 (1.0%)	1,873 (0.9%)
Moderate or severe renal disease	3,617 (5.2%)	8,385 (3.9%)
Hemiplegia	1,961 (2.8%)	4,644 (2.1%)
HIV/AIDS	214 (0.3%)	419 (0.2%)
Liver disease	1,424 (2.0%)	3,639 (1.7%)
* Mild liver disease*	1,080 (1.6%)	2,892 (1.3%)
* Moderate or severe liver disease*	344 (0.5%)	747 (0.3%)
Diabetes	13,875 (19.9%)	43,306 (19.9%)
* Diabetes without end-organ damage*	10,686 (15.3%)	34,867 (16%)
* Diabetes with end-organ damage*	3,189 (4.6%)	8,439 (3.9%)
Cancer (except non melanoma skin cancer)	8,794 (12.6%)	29,383 (13.5%)
* Non metastatic cancer (including lymphoma and leukemia)*	7,827 (11.2%)	26,900 (12.4%)
* Metastatic solid tumor*	967 (1.4%)	2,483 (1.1%)
Charlson index		
0	37,769 (54.1%)	121,987 (56.1%)
1	12,377 (17.7%)	39,082 (18.0%)
≥ 2	19,640 (28.1%)	56,508 (26.0%)
Treatment duration (in days)		
Median [Q1–Q3]	66 [29–182]	87 [31–220]
Range	1–1826	1–1825
Events		
First suicidal events	52 (0.07%)	133 (0.06%)
Suicide deaths	18 (0.03%)	47 (0.02%)
First self-harm hospitalisation	34 (0.05%)	87 (0.04%)
First self-harm hospitalisation, with violent means*	11 (0.02%)	21 (0.01%)
First self-harm hospitalisation, with admission to an intensive care unit	13 (0.02%)	16 (0.01%)
First self-harm hospitalisation, with violent means* and intensive care unit	3 (0.004%)	4 (0.002%)
Treatment duration up to suicide death (in days)		
Median [Q1–Q3]	36 [8–36]	129 [27–289]
Range	2–435	3–1,393
Treatment duration up to first self-harm hospitalisation (in days)		
Median [Q1–Q3]	73 [23–138]	101 [29–265]
Range	3–1017	2–918

*Self-harm with a violent means defined by an ICD-10 code (X66-X83).

HIV/AIDS: human immunodeficiency virus infection; acquired immunodeficiency syndrome.

[Q1-Q3]: first and fourth quartiles.

Suicidal events occurred for 52 individuals (0.07%) exposed to finasteride, including 18 deaths (0.03%) and 34 first self-harm hospitalisations (0.05%), and for 133 individuals (0.06%) exposed to dutasteride, including 47 deaths (0.02%) and 87 first self-harm hospitalisations (0.04%). Only one individual (in the dutasteride group) had a hospitalisation for self-harm before suicide death during the follow-up. Among individuals who were hospitalised for self-harm during the follow-up, 11 (0.02%) of the self-harm events were performed by a violent means in the finasteride group vs. 21 (0.01%) in the dutasteride group and 13 (0.02%) required admission to an intensive care unit vs. 16 (0.01%) in the dutasteride group.

A series of unadjusted analyses are presented in the supplemental material (Kaplan–Meier curves in Online Appendix 4, incidence rates of events in Online Appendix 5, crude associations with the composite outcome for covariates in the whole study population in Online Appendix 6, and unadjusted risks of outcome according to the study population in Online Appendix 7).

The distributions of stabilized weights of propensity scores according to the study population for the IPTW adjusted analyses are presented in Online Appendix 8. In all analyses (except those restricted to individuals with a history of self-harm hospitalisation), the stabilized weights had a mean of 1 and their maximal individual value was lower than 2.5, and thus did not suggest misspecification of the treatment model or lack of positivity. In the analyses conducted on the whole study population, the standardized differences were already < 10% (the threshold generally considered to be negligible) before IPTW for almost all covariates and were almost null for all covariates after IPTW.

In the IPTW adjusted analyses (Table 2 and Online Appendix 5), finasteride treatment was not associated with the composite outcome for the whole study population (52 outcomes for 31,344.9 person-years for finasteride vs. 133/110,329.4 person-years for dutasteride, HR = 1.21 [95%CI = 0.87–1.67]). When restricting the analysis to sub-populations according to psychiatric history, the association was not statistically significant (notably, not for individuals without psychiatric disorders), except for individuals with a history of mood disorders (25/4,012.2 person-years for finasteride vs. 46/11,876.5 person-years for dutasteride; HR = 1.64 [95%CI = 1.00–2.68], *p* = 0.049).

**Table 2:** Risk of suicide death and self-harm hospitalisation (considered separately) associated with finasteride compared to that with dutasteride (IPTW adjusted analyses).

Analysed population	Number of events (incidence rate /1000 person-years)		HR [95% CI]	*p* value
	Finasteride	Dutasteride		
Outcome: suicide death or self-harm hospitalisation				
Whole study population	52 (1.66)	133 (1.21)	1.21 [0.87;1.67]	0.252
Whole study population, censoring at 90 days maximum	32 (2.74)	64 (1.64)	1.46 [0.95;2.25]	0.081
Excluding patients with a history of psychiatric disorders or self-harm	12 (0.55)	49 (0.60)	0.78 [0.41;1.47]	0.437
Restricted to patients with a history of psychiatric disorders or self-harm	40 (4.29)	84 (2.97)	1.39 [0.95;2.03]	0.090
Restricted to patients with a history of self-harm within 3 years	6 (75.1)	14 (66.9)	1.54 [0.55;4.31]	0.408
Restricted to patients with a history of mood disorders	25 (6.23)	46 (3.87)	1.64 [1.00;2.68]	0.049
Restricted to patients with a history of anxiety disorders	26 (5.40)	51 (3.42)	1.49 [0.92;2.41]	0.101
Restricted to patients with a history of other psychiatric disorders	25 (5.09)	55 (3.93)	1.26 [0.78;2.04]	0.345
Restricted to patients with a history of mood disorders, censoring at 90 days	14 (9.50)	24 (5.59)	1.70 [0.88;3.31]	0.116
Outcome: suicide death				
Whole study population	18 (0.57)	47 (0.43)	1.25 [0.72;2.16]	0.432
Whole study population, censoring at 90 days maximum	12 (1.03)	22 (0.56)	1.56 [0.77;3.17]	0.215
Excluding patients with a history of psychiatric disorders or self-harm	5 (0.23)	21 (0.26)	0.77 [0.29;2.05]	0.600
Restricted to patients with a history of psychiatric disorders or self-harm	13 (1.39)	26 (0.92)	1.56 [0.80;3.06]	0.192
Restricted to patients with a history of self-harm within 3 years	0 (0.00)	1 (4.74)	NE	
Restricted to patients with a history of mood disorders	8 (1.99)	10 (0.84)	2.71 [1.07;6.91]	0.036
Restricted to patients with a history of anxiety disorders	8 (1.66)	14 (0.94)	1.79 [0.75;4.28]	0.192
Restricted to patients with a history of other psychiatric disorders	9 (1.83)	15 (1.07)	1.79 [0.78;4.10]	0.167
Restricted to patients with a history of mood disorders, censoring at 90 days	3 (2.04)	5 (1.16)	4.66 [1.10;19.7]	0.037
Outcome: self-harm hospitalisation				
Whole study population	34 (1.08)	87 (0.79)	1.17 [0.79;1.75]	0.436
Whole study population, censoring at 90 days maximum	20 (1.71)	43 (1.10)	1.37 [0.80;2.35]	0.246
Excluding patients with a history of psychiatric disorders or self-harm	7 (0.32)	28 (0.34)	0.78 [0.34;1.80]	0.566
Restricted to patients with a history of psychiatric disorders or self-harm	27 (2.89)	59 (2.09)	1.29 [0.81;2.04]	0.282
Restricted to patients with a history of self-harm within 3 years	6 (75.2)	13 (62.1)	1.68 [0.60;4.75]	0.326
Restricted to patients with a history of mood disorders	17 (4.24)	36 (3.03)	1.34 [0.75;2.41]	0.321
Restricted to patients with a history of anxiety disorders	18 (3.74)	38 (2.55)	1.34 [0.76;2.37]	0.315
Restricted to patients with a history of other psychiatric disorders	16 (3.26)	40 (2.86)	1.06 [0.59;1.92]	0.840
Restricted to patients with a history of mood disorders, censoring at 90 days	9 (6.11)	21 (4.89)	1.25 [0.57;2.75]	0.583

Cox proportional hazards models controlled by IPTW for the following potential confounders: age, year of treatment initiation, pre-existing prostatic and psychiatric conditions and treatments, comorbidities used in the Charlson index.

CI: confidence interval.

HR: hazard ratio.

IPTW: inverse probability of treatment weighting.

NE: not estimable.

When considering suicide deaths only as the outcome (Table 2 and Online Appendix 5), finasteride treatment was not statistically associated with suicide death in the whole population, particularly among individuals without an identified psychiatric history. However, it was associated with an increased risk in the population of individuals with a history of mood disorders (8/4,017.4 person-years for finasteride vs. 10/11,880.4 person-years for dutasteride, HR = 2.71 [95%CI = 1.07–6.91] when the entire duration of treatment was considered).

When considering self-harm hospitalisations of all severity as outcomes (Table 2), finasteride was not associated with an increased risk in the whole study population or in the sub-populations. When considering severe self-harm hospitalisations only as outcomes (Table 3 and Online Appendix 5), finasteride was associated with an increased risk of self-harm by a violent means (first definition of severe self-harm hospitalisations) for individuals with a history of mood disorders only, but only when considering the entire duration of treatment in the analyses (6/4,012.2 person-years for finasteride vs. 6/11,876.5 person-years for dutasteride; HR = 3.11 [95%CI = 1.01–9.61]). Finasteride was associated with an increased risk of self-harm with admission to an intensive care unit (second definition of severe self-harm hospitalisations) in the whole study population (13/31,344.9 person-years for finasteride vs. 16/110,329.4 person-years for dutasteride, HR = 2.60 [95%CI = 1.25–5.38]) and for individuals with any psychiatric history (particularly for individuals with a history of mood or anxiety disorders), but not for individuals without a psychiatric history.

**Table 3:** Risk of self-harm by a violent means or admission to an intensive care unit associated with finasteride compared to that with dutasteride (IPTW adjusted analyses).

Analysed population	Number of events (incidence rate /1000 person-years)		HR [95% CI]	*p* value
	Finasteride	Dutasteride		
Outcome: self-harm with violent means*				
Whole study population	11 (0.35)	21 (0.19)	1.75 [0.84;3.64]	0.134
Whole study population, censoring at 90 days maximum	5 (0.43)	8 (0.20)	2.07 [0.67;6.35]	0.205
Excluding patients with a history of psychiatric disorders or self-harm	3 (0.14)	11 (0.13)	0.96 [0.27;3.42]	0.944
Restricted to patients with a history of psychiatric disorders or self-harm	8 (0.86)	10 (0.35)	2.38 [0.94;6.05]	0.068
Restricted to patients with a history of self-harm within 3 years	1 (12.5)	2 (9.55)	0.96 [0.09;10.7]	0.975
Restricted to patients with a history of mood disorders	6 (1.50)	6 (0.51)	3.11 [1.01;9.61]	0.049
Restricted to patients with a history of anxiety disorders	5 (1.04)	5 (0.34)	3.03 [0.86;10.6]	0.084
Restricted to patients with a history of other psychiatric disorders	4 (0.81)	7 (0.50)	1.72 [0.50;5.99]	0.393
Restricted to patients with a history of mood disorders, censoring at 90 days	3 (2.04)	4 (0.93)	2.35 [0.53;10.5]	0.263
Outcome: self-harm with admission to an intensive care unit				
Whole study population	13 (0.41)	16 (0.15)	2.60 [1.25;5.38]	0.010
Whole study population, censoring at 90 days maximum	8 (0.68)	6 (0.15)	4.32 [1.51;12.4]	0.006
Excluding patients with a history of psychiatric disorders or self-harm	4 (0.18)	7 (0.09)	1.87 [0.55;6.30]	0.314
Restricted to patients with a history of psychiatric disorders or self-harm	9 (0.96)	9 (0.32)	3.05 [1.21;7.69]	0.018
Restricted to patients with a history of self-harm within 3 years	3 (37.6)	3 (14.3)	4.15 [0.75;23.1]	0.104
Restricted to patients with a history of mood disorders	7 (1.74)	5 (0.42)	3.97 [1.26;12.5]	0.018
Restricted to patients with a history of anxiety disorders	7 (1.45)	4 (0.27)	5.46 [1.61;18.6]	0.007
Restricted to patients with a history of other psychiatric disorders	5 (1.02)	8 (0.57)	1.70 [0.55;5.26]	0.354
Restricted to patients with a history of mood disorders, censoring at 90 days	4 (2.71)	1 (0.23)	11.4 [1.29;100.1]	0.029

Cox proportional hazards models controlled by IPTW for the following potential confounders: age, year of treatment initiation, pre-existing prostatic and psychiatric conditions and treatments, comorbidities used in the Charlson index.

*Self-harm with a violent means defined by an ICD-10 code (X66-X83).

CI: confidence interval.

HR: hazard ratio.

IPTW: inverse probability of treatment weighting.

Sensitivity analyses with further adjusting of the IPTW analyses for age as a time-varying covariate yielded relatively similar results, with risks of the composite outcome associated with finasteride being more frequently significant than in the main analyses. In addition to the populations in which the risk was significantly increased in the main analysis, it was also significantly increased for individuals with a psychiatric history (particularly mood and anxiety disorders, Online Appendix 9).

## Discussion

Our study found no increased risk of suicide death or self-harm hospitalisation with finasteride relative to dutasteride in a large population of men treated for benign prostatic hyperplasia. However, we found finasteride to be associated with an increased risk of suicide death or severe self-harm (as defined by the use of a violent suicidal means or admission to an intensive care unit) for patients with a history of mood disorders relative to that for dutasteride. These results were, however, based on a low number of events and should thus be interpreted with caution.

The association between finasteride exposure and suicidal behaviour is currently subject to scientific debate, notably on whether this association is attributable to finasteride itself, sexual adverse events secondary to finasteride treatment, or the disease treated with finasteride (particularly in the case of androgenetic alopecia)^52,53^. Our study was conducted on men treated for BPH, not for androgenetic alopecia. BPH is a disease that can also negatively affect the quality of life and be associated with depressive symptoms^32^, but such an impact may be lower than that of androgenetic alopecia in younger, treatment-seeking men. Furthermore, the two drugs considered, finasteride and dutasteride, are both indicated as a second-line treatment for patients with BPH and are thus indicated at the same stage of the disease. Finally, finasteride and dutasteride are known to show similar efficacy on lower urinary tract symptoms and to have a similar sexual safety profile^29^. Indeed, some investigators even found less frequent sexual adverse events among men treated with finasteride than those treated with dutasteride^54^. Overall, these settings tended to reduce the risk of confounding in the comparison between finasteride users and dutasteride users. Previous studies on the suicidal risk of finasteride did not compare it with dutasteride or considered exposure to 5α-reductase inhibitors as a whole^8,55^. Welk et al. followed-up individuals from the initiation of 5α-reductase inhibitor treatment and allowed patients to switch from one 5α-reductase inhibitor to another. They did not find any difference between finasteride and dutasteride when assessing the difference in suicidal risk according to the drug used for the first prescription^6^. In our study, individuals who switched among 5α-reductase inhibitor medications were censored at treatment switch.

Although depression is a well-known major risk factor for suicide^49^, most people with depression will not attempt suicide or die by suicide. Multifactorial causes are involved in the occurrence of a suicidal state and in the transition from suicidal ideas to a suicidal act. Our finding that the effect of finasteride on suicidal risk, if any, would be more apparent in people with a history of mood disorders suggests that finasteride alone is unlikely to facilitate a suicidal act. Of note, a recent study found genetic evidence that inhibition of 5α-reductase was associated with depression^56^. However, for reasons largely unknown at this stage, finasteride may further trigger a suicidal act in those who are already in a fragile mental state or at-risk to be in such a mental state.

Biological evidence on the impact of finasteride on suicidal behaviour is very scarce^57^. Biological studies have instead investigated the mechanisms involving finasteride or 5α-reductase inhibitors in the development of depression, including alterations in neuro-steroid levels (notably allopregnanolone), dopaminergic dysfunction, reduced hippocampal neurogenesis, increased neuro-inflammation, alteration of the hypothalamic–pituitary–adrenal axis, and epigenetic modifications^58^. The increased risk of suicide death or severe self-harm among men with previous mood disorders treated with finasteride relative to those treated with dutasteride could be explained both by an increased suicidal risk with finasteride, as well as a protective effect of dutasteride^59^. It is not possible to distinguish between these two hypotheses with the data used for our study. Finasteride is known to cross the blood–brain barrier and thus can affect concentrations of neuro-steroids and their metabolites in the cerebrospinal fluid^16–18,60–62^. It is not currently clear to what extent dutasteride also crosses the blood–brain barrier and further biological investigations are needed to address this issue, as such a difference could explain the difference observed in our study between two drugs of the same pharmacological class. It cannot be excluded that dutasteride is also associated with an increased suicidal risk for at-risk individuals.

It should be noted that the association with suicide death or self-harm with admission to an intensive care unit was stronger among men with an identified history of mood disorders during the first 90 days of exposure, which could be consistent with a trigger effect of treatment initiation. However, it should also be noted that the observed stronger risk of a severe outcome during the early weeks of treatment was not observed for hospitalisations for self-harm by violent means. Generally, the number of events observed in the analyses restricted to populations with psychiatric comorbidities was low and caution must be applied in their interpretation. Another important contribution of our work is the identification of men with prior psychiatric disorders, particularly mood disorders, as being a possible at-risk population. In the study of Welk et al., no statistically significant interaction was found for suicide between the use of 5α-reductase inhibitors and a history of depression^6^. In light of these results, screening for depression before treatment initiation could have been considered as not being necessary^24^. Our study, by contrast, suggests the potential relevance of such a screening before the initiation of 5α-reductase inhibitor.

Large population-wide studies have not supported a long-term effect of 5α-reductase inhibitors on suicidal events^6,9^. However, concerning the well-documented persistent sexual adverse events in the literature, it cannot be excluded that a risk of suicidal behaviour may persist after the first weeks of treatment or after treatment discontinuation^10–12,29,54,63–68^. Our study compared finasteride 5 mg with dutasteride 0.5 mg, both indicated for the treatment of BPH, and was not designed to assess the suicidal risk of 5α-reductase inhibitors compared with other treatments. Finasteride is also indicated for the treatment of androgenetic alopecia at a five-fold lower daily dosage of 1 mg^69^. Although our results cannot be directly generalized to men treated for androgenetic alopecia with finasteride 1 mg, it cannot be excluded that the increased risk of severe suicidal behaviour observed in our study in men with mood disorders treated for BPH may also exist for finasteride 1 mg.

Our study took full advantage of the SNDS database^35^. This nationwide database comprises prospectively and systematically collected comprehensive information on claims for inpatient and outpatient care linked to the national causes-of-death registry database. This allowed us to study linked data on exposure (drug delivery) and outcomes (causes of death and hospitalisation diagnoses) for a large population of nearly 300,000 men initiating treatment with a 5α-reductase inhibitor for BPH. The accuracy of the information available on hospital discharges in the SNDS also allowed further examination of self-harm hospitalisations with self-harm by violent means and self-harm with admission to an intensive care unit. These analyses helped us to understand the discrepant results obtained for suicide deaths and self-harm hospitalisations, all severities considered, and were consistent in terms of the association with severe suicidal behaviour in men with mood disorders. Self-harm and suicide death are two very different behaviours from a sociodemographic point of view: suicide deaths are more frequent among older men, whereas self-harming behaviours are more frequent among younger women^49,70^. They are also very different from a biological point of view^71^, notably with biological evidence of a correlation between dysregulation of the hypothalamic–pituitary–adrenal axis and the violence of self-harming behaviour^72^.

We followed an active comparator, new user study design, which allows mitigation of the immortal time bias and the assessment of a trigger effect of treatment initiation, which cannot be explored with a prevalent user design^9,73^. It also allows the mitigation of confounding by indication by implicitly controlling for indication^33^, as both finasteride and dutasteride are indicated as a second line treatment for BPH. Individuals included in the two groups already had highly similar baseline characteristics before IPTW (Online Appendix 8). Given the low number of suicidal events during the follow-up in the study population, from a statistical point of view, classic adjustment for covariates would have required selecting a restricted number of potential confounders to be controlled for, whereas IPTW allows controlling for a larger number of covariates. Finally, stabilized weight distributions did not suggest misspecification of the treatment model or lack of positivity in any of the analyses. Residual unmeasured confounding could have persisted with the lack of precise sociodemographic factors in the SNDS, but these factors appear to be rather unlikely to confound the association between suicidal behaviour and the choice of BPH treatment between finasteride and dutasteride.

Psychiatric diseases are known to be difficult to identify in the SNDS database^74^. They were identified in the year prior to treatment initiation using hospitalisation or LTD diagnostic codes. They could also be identified by the delivery of psychotropic drugs at three different dates during the year prior to treatment initiation. The lists of codes used for this study, submitted for expert review, are commonly used, notably in the SNDS mapping tool developed by the French National Health Insurance^75^. For individuals for whom the psychiatric condition was identified solely on the basis of drug delivery, the drug had thus to be delivered at three different dates, limiting the over-diagnosis of individuals treated punctually. In addition, ICD-10 codes X60 to X84 do not allow a distinction between non-suicidal self-injuries and suicide attempts among self-harm hospitalisations^38^. However, only acts leading to hospitalisation were recorded and extracted here, i.e. the most severe and life-threatening acts. Based on these definitions, men on finasteride more frequently had pre-existing psychiatric conditions and mood disorders as compared to those on dutasteride. Although these differences were taken into account in the IPTW adjusted analyses, we cannot exclude residual confounding due to failure to fully identify past psychiatric disorders and account for their severity based on information available in the SNDS.

On another note, as finasteride is also marketed with the indication of androgenetic alopecia unlike dutasteride, some prescribers may be more inclined to use finasteride than dutasteride in men with androgenetic alopecia, even if the dosage is lower and the brand name different for low-dose finasteride in the treatment of androgenetic alopecia. Hair loss might thus be more frequent in the finasteride group, introducing confounding in the observed association between finasteride and suicidal behaviour. Unfortunately, adjustment for androgenetic alopecia cannot be performed with our data, as this information in not available in SNDS. Though, several elements tend to mitigate this risk of bias. Firstly, the impact of hair loss on quality of life is lower in older men (as in our study population) than in younger men (treated with finasteride 1 mg), notably when hair loss occurred earlier^76,77^. Secondly, dutasteride has also been described as an efficient treatment for androgenetic alopecia^31^, and some prescribers may know this common feature between the two drugs. However, such a bias, if any, would lead to an overestimation of the risk associated with finasteride. This provides a further argument for caution in interpreting the observed association in men with a history of mood disorders.

A final limitation of our study was that we had to restrict the study population to health insurance general scheme beneficiaries. Data on medical causes of death, which were used for the identification of suicide deaths, are indirectly and deterministically linked to claims data by using several variables common to the databases, most importantly the date of death, for which comprehensive data in the SNDS is only available during the study period for general scheme beneficiaries. The general scheme covers more than three quarters of the population living in France (employed, unemployed, retired, and disadvantaged people). Certain occupational groups are covered by specific health insurance schemes, mainly farmers and self-employed workers, all reimbursing all medical care at the same rate, thus limiting differences in accessibility to healthcare between schemes. Farmers (who represent 5% of the population living in France, and the second largest health insurance scheme)^35^ are known to be older than the general population and at a higher risk of suicide death than other occupational groups in France^78,79^. Nevertheless, the biological features that could explain the association observed between finasteride and severe suicidal behaviour in men with mood disorders may be no different between general scheme beneficiaries and farmers or beneficiaries of other health insurance schemes. Including them, if the linkage of the causes of death was as good as for general scheme beneficiaries, would, however, have helped increase statistical power. This highlights the necessity of improving the linkage of causes-of-death data in the SNDS using the same unique anonymous number used for the linkage of outpatient claims and hospital discharge data to eliminate the necessity of comprehensive information on the date of death.

## Conclusions

Our study showed no increased risk of suicidal behaviour in the general population of men treated for BPH with finasteride relative to those treated with dutasteride, particularly, among men without psychiatric disorders. However, among men with a history of mood disorders, we found that finasteride may be associated with a higher risk of suicide death and severe self-harm with the use of violent means or admission to an intensive care unit than dutasteride. Considering the low number of violent outcomes observed among individuals with mood disorders, the results concerning this specific population need to be confirmed with other epidemiological studies in countries in which national healthcare data allow them. Further research is also needed to investigate the putative risk for younger men treated with low-dose finasteride for androgenetic alopecia. Moreover, the biological mechanisms involved that could explain the suicidal risk associated with this medication will have to be further investigated.

## Supplementary Information

Supplementary Information.

## Data Availability

Data from the SNDS used in this work cannot be shared by the Authors due to French laws restricting access to health data (https://www.snds.gouv.fr/). Applications to access the SNDS must be submitted to the French Health Data Hub (https://www.health-data-hub.fr/).

## Abbreviations

**95****% CI**: 95% Confidence interval
**ATC**: Anatomical therapeutic chemical classification system
**BPH**: Benign prostatic hyperplasia
**CCAM**: *Classification Commune des Actes Médicaux* (French medical classification for clinical procedures)
**HR**: Hazard ratio
**ICD-10**: International statistical classification of diseases and related health problems, tenth revision
**IPTW**: Inverse probability of treatment weighting
**LTD**: Long-term disease
**PMSI**: *Programme de Médicalisation des Systèmes d’Information* (French national hospital discharge database)
**Q1–Q3**: First and third quartiles
**SNDS**: *Système National des Données de Santé* (French national health data system)
**SNIIRAM**: *Système National d’Information Inter-Régimes de l’Assurance Maladie* (French national health insurance claims information system)
**US**: United States
