# Supplementary material for: Suicidal risk associated with finasteride versus dutasteride among men treated for benign prostatic hyperplasia: nationwide cohort study
Source: Sci Rep. 2023 Mar 31;13:5308. doi: 10.1038/s41598-023-32356-3 (PMC10066399; doi:10.1038/s41598-023-32356-3)
Supplement: Supplementary file 1 — Supplementary Information. [file 41598_2023_32356_MOESM1_ESM.pdf]

Suicidal risk associated with finasteride versus dutasteride among men treated for benign prostatic hyperplasia: Nationwide cohort study

Moussa LAANANI, Alain WEILL, Fabrice JOLLANT, Mahmoud ZUREIK, and Rosemary DRAY-SPIRA

ADDITIONAL FILE

Content:

Appendix 1. List of ICD-10 codes for suicide cause of death and self-harm hospitalisation

Appendix 2. List of codes for pre-existing psychiatric conditions and treatments

Appendix 3. List of codes for pre-existing prostatic conditions and treatments

Appendix 4. Kaplan-Meier curves for the main analysis (entire cohorts of finasteride or dutasteride new users followed up to event, end of treatment exposure, or censoring)

Appendix 5. Number of exposed individuals, events, and incidence rates for each analysis

Appendix 6. Crude associations with suicide death or self-harm hospitalisation (composite outcome) for variables included in the IPTW treatment model

Appendix 7. Risk of suicide death or self-harm hospitalisation associated with finasteride relative to dutasteride exposure (unadjusted Cox proportional hazards models)

Appendix 8. Propensity score stabilized weight distribution in IPTW adjusted analyses

Appendix 9. Risk of suicide death or self-harm hospitalisation associated with finasteride relative to dutasteride exposure (IPTW adjusted analyses, adjusting for age as a time-varying covariate)

Appendix 1. List of ICD-10 codes for suicide cause of death and self-harm hospitalisation

ICD-10 code	Description
**X60**	Intentional self-poisoning by and exposure to nonopioid analgesics, antipyretics and antirheumatics
**X61**	Intentional self-poisoning by and exposure to antiepileptic, sedative-hypnotic, antiparkinsonism and psychotropic drugs, not elsewhere classified
**X62**	Intentional self-poisoning by and exposure to narcotics and psychodysleptics [hallucinogens], not elsewhere classified
**X63**	Intentional self-poisoning by and exposure to other drugs acting on the autonomic nervous system
**X64**	Intentional self-poisoning by and exposure to other and unspecified drugs, medicaments and biological substances
**X65**	Intentional self-poisoning by and exposure to alcohol
**X66**	Intentional self-poisoning by and exposure to organic solvents and halogenated hydrocarbons and their vapours
**X67**	Intentional self-poisoning by and exposure to other gases and vapours
**X68**	Intentional self-poisoning by and exposure to pesticides
**X69**	Intentional self-poisoning by and exposure to other and unspecified chemicals and noxious substances
**X70**	Intentional self-harm by hanging, strangulation and suffocation
**X71**	Intentional self-harm by drowning and submersion
**X72**	Intentional self-harm by handgun discharge
**X73**	Intentional self-harm by rifle, shotgun and larger firearm discharge
**X74**	Intentional self-harm by other and unspecified firearm discharge
**X75**	Intentional self-harm by explosive material
**X76**	Intentional self-harm by smoke, fire and flames
**X77**	Intentional self-harm by steam, hot vapours and hot objects
**X78**	Intentional self-harm by sharp object
**X79**	Intentional self-harm by blunt object
**X80**	Intentional self-harm by jumping from a high place
**X81**	Intentional self-harm by jumping or lying before moving object
**X82**	Intentional self-harm by crashing of motor vehicle
**X83**	Intentional self-harm by other specified means
**X84**	Intentional self-harm by unspecified means

ICD-10: International Classification of Diseases, 10th revision

Appendix 2. List of codes for pre-existing psychiatric conditions and treatments

Code	Description
**Depression and mood disorders**	
ICD-10 codes (hospitalisation or LTD diagnoses)	
**F30**	Manic episode
**F31**	Bipolar affective disorder
**F32**	Depressive episode
**F33**	Recurrent depressive disorder
**F34**	Persistent mood [affective] disorders
**F38**	Other mood [affective] disorders
**F39**	Unspecified mood [affective] disorder
ATC codes (drug delivery)	
**N06A**	Antidepressants (excluding Levotonine® oxitriptan: N06AX01)
**N05AN01**	Lithium
**N03AG01**	Valproic acid (Dépakote® only)
**N03AG02**	Valpromide (Dépamide®)
**Anxiety**	
ICD-10 codes (hospitalisation or LTD diagnoses)	
**F40**	Phobic anxiety disorders
**F41**	Other anxiety disorders
**F42**	Obsessive-compulsive disorder
**F43**	Reaction to severe stress, and adjustment disorders
**F44**	Dissociative [conversion] disorders
**F45**	Somatoform disorders
**F48**	Other neurotic disorders
ATC codes (drug delivery)	
**N05B**	Anxiolytics (excluding Mepronizine® meprobamate combination: N05BC51)
**Other psychiatric diagnoses and treatments**	
ICD-10 codes (hospitalisation or LTD diagnoses)	
**F0**	Organic, including symptomatic, mental disorders
**F1**	Mental and behavioural disorders due to psychoactive substance use
**F2**	Schizophrenia, schizotypal and delusional disorders
**F5**	Behavioural syndromes associated with physiological disturbances and physical factors
**F6**	Disorders of adult personality and behaviour
**F7**	Mental retardation
**F8**	Disorders of psychological development
**F90-F98**	Behavioural and emotional disorders with onset usually occurring in childhood and adolescence
**F99**	Unspecified mental disorder
ATC codes (drug delivery)	
**N05A**	Antipsychotics (excluding lithium N05AN01, veralipride N05AL06, chlorproethazine N05AA07)
**N05BC51**	Meprobamate combination (Mepronizine®)
**N05C**	Hypnotics and sedatives (excluding midazolam N05CD08, melatonin N05CH01, scopolamine N05CM05, Valerianae radix N05CM09)*

ATC: Anatomical Therapeutic Chemical classification system
ICD-10: International Classification of Diseases, 10th revision
LTD: long-term disease

Appendix 3. List of codes for pre-existing prostatic conditions and treatments

Code	Description
**Transurethral resection of the prostate**	
CCAM codes (procedures performed in hospital)	
**JGFA014**	Palliative resection of the prostate [Urethral recalibration], by urethrocystoscopy
**JGFA015**	Resection of a prostatic hyperplasia, by urethrocystoscopy
**JGFA016**	Resection or marsupialization of a prostate collection or urethral diverticulum, by urethrocystoscopy
**Alpha-blockers**	
ATC codes (drug delivery)	
**G04CA01**	Alfuzosin
**G04CA02**	Tamsulosin
**G04CA03**	Terazosin
**G04CA04**	Silodosin
**G04CA**	Doxazosin
**G04CA52**	Tamsulosin (in combination with dutasteride)
**Prostate cancer**	
ICD-10 codes (hospitalisation or LTD diagnoses)	
**C61**	Malignant neoplasm of prostate
**D075**	Carcinoma in situ of prostate

ATC: Anatomical Therapeutic Chemical classification system
CCAM: *Classification Commune des Actes Médicaux* (French medical classification for clinical procedures)
ICD-10: International Classification of Diseases, 10th revision
LTD: long-term disease

Appendix 4. Kaplan-Meier curves for the main analysis (entire cohorts of finasteride or dutasteride new users followed up to event, end of treatment exposure, or censoring)

On the left, graphs representing the survival curves for the whole follow-up period; on the right, graphs focusing on the first three months. Note that the y axis does not have the same minimum between graphs.

Self-harm hospitalisation and suicide deaths (composite event)

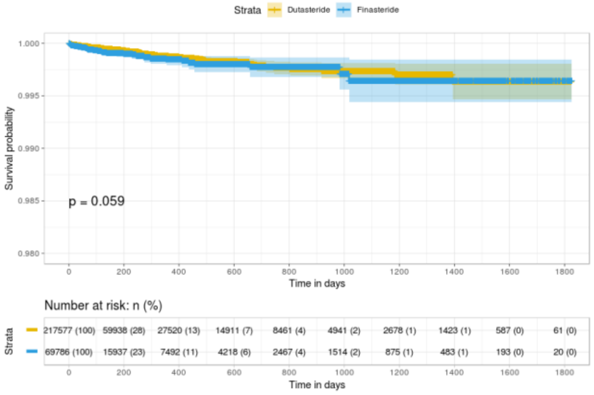

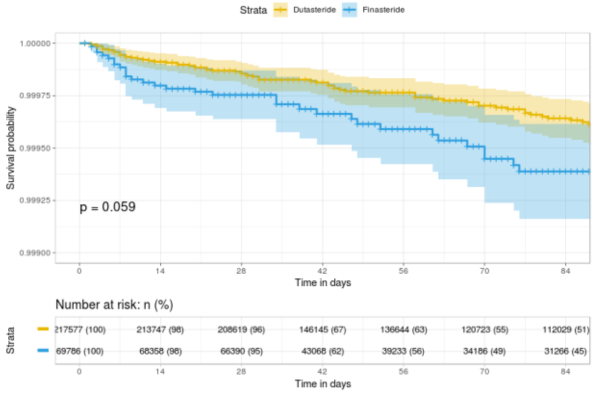

Suicide deaths

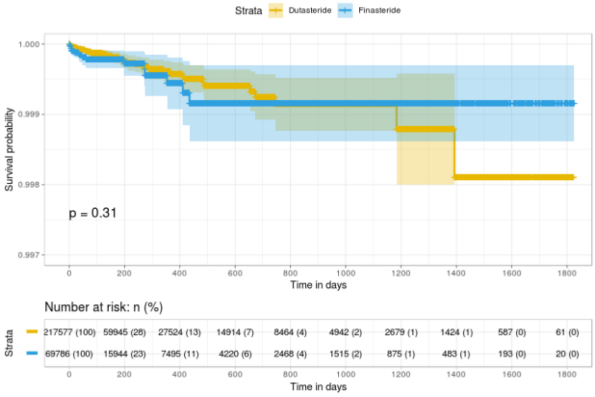

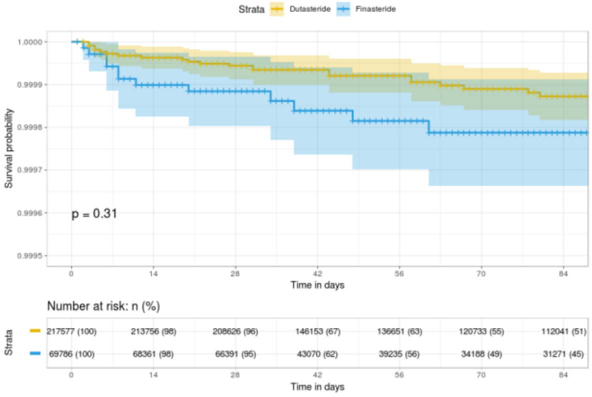

Self-harm hospitalisation

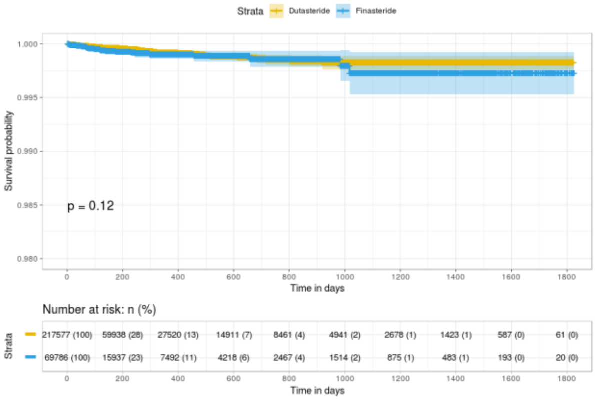

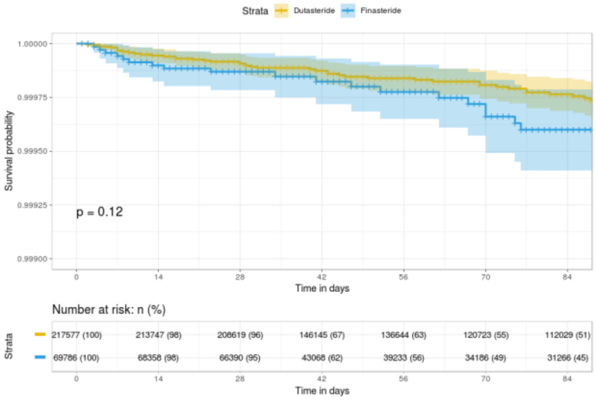

Appendix 5. Number of exposed individuals, events, and incidence rates for each analysis

Analysed population	Exposed to finasteride			Exposed to dutasteride		
	Number exposed	Events / person-years	Incidence rate*	Number exposed	Events / person-years	Incidence rate*
Outcome: suicide death or self-harm hospitalisation						
Whole study population	69,786	52/31,344.9	1.66	217,577	133/110,329.4	1.21
Whole study population, censoring at 90 days maximum	69,786	32/11,689.5	2.74	217,577	64/39,082.2	1.64
Excluding patients with a history of psychiatric disorder or self-harm	49,962	12/22,013.3	0.55	161,993	49/82,073.9	0.60
Restricted to patients with a history of psychiatric disorder or self-harm	19,824	40/9,331.6	4.29	55,584	84/28,255.5	2.97
Restricted to patients with a history of self-harm within 3 years	185	6/79.8	75.16	466	14/209.4	66.86
Restricted to patients with a history of mood disorders	8,638	25/4,012.2	6.23	23,503	46/11,876.5	3.87
Restricted to patients with a history of anxiety disorders	10,371	26/4,813.7	5.40	29,099	51/14,890.8	3.42
Restricted to patients with a history of other psychiatric disorder	10,413	25/4,910.2	5.09	27,989	55/13,989.4	3.93
Restricted to patients with a history of mood disorders, censoring at 90 days	8,638	14/1,473.6	9.50	23,503	24/4,296.0	5.59
Outcome: suicide death						
Whole study population	69,786	18/31,352.8	0.57	217,577	47/110,343.1	0.43
Whole study population, censoring at 90 days maximum	69,786	12/11,690.1	1.03	217,577	22/39,084.2	0.56
Excluding patients with a history of psychiatric disorder or self-harm	49,962	5/22,014.7	0.23	161,993	21/82,079.5	0.26
Restricted to patients with a history of psychiatric disorder or self-harm	19,824	13/9,338.1	1.39	55,584	26/28,263.6	0.92
Restricted to patients with a history of self-harm within 3 years	185	0/82.2	0.00	466	1/210.8	4.74
Restricted to patients with a history of mood disorders	8,638	8/4,017.4	1.99	23,503	10/11,880.4	0.84
Restricted to patients with a history of anxiety disorders	10,371	8/4,817.5	1.66	29,099	14/14,894.2	0.94
Restricted to patients with a history of other psychiatric disorder	10,413	9/4,913.9	1.83	27,989	15/13,996.3	1.07
Restricted to patients with a history of mood disorders, censoring at 90 days	8,638	3/1,473.8	2.04	23,503	5/4,297.1	1.16
Outcome: self-harm hospitalisation						
Whole study population	69,786	34/31,344.9	1.08	217,577	87/110,329.4	0.79
Whole study population, censoring at 90 days maximum	69,786	20/11,689.5	1.71	217,577	43/39,082.2	1.10
Excluding patients with a history of psychiatric disorder or self-harm	49,962	7/22,013.3	0.32	161,993	28/82,073.9	0.34
Restricted to patients with a history of psychiatric disorder or self-harm	19,824	27/9,331.6	2.89	55,584	59/28,255.5	2.09
Restricted to patients with a history of self-harm within 3 years	185	6/79.8	75.16	466	13/209.4	62.09
Restricted to patients with a history of mood disorders	8,638	17/4,012.2	4.24	23,503	36/11,876.5	3.03
Restricted to patients with a history of anxiety disorders	10,371	18/4,813.7	3.74	29,099	38/14,890.8	2.55
Restricted to patients with a history of other psychiatric disorder	10,413	16/4,910.2	3.26	27,989	40/13,989.4	2.86
Restricted to patients with a history of mood disorders, censoring at 90 days	8,638	9/1,473.6	6.11	23,503	21/4,296.0	4.89
Outcome: self-harm hospitalisation with violent means**						
Whole study population	69,786	11/31,344.9	0.35	217,577	21/110,329.4	0.19
Whole study population, censoring at 90 days maximum	69,786	5/11,689.5	0.43	217,577	8/39,082.2	0.20
Excluding patients with a history of psychiatric disorder or self-harm	49,962	3/22,013.3	0.14	161,993	11/82,073.9	0.13
Restricted to patients with a history of psychiatric disorder or self-harm	19,824	8/9,331.6	0.86	55,584	10/28,255.5	0.35
Restricted to patients with a history of self-harm within 3 years	185	1/79.8	12.53	466	2/209.4	9.55
Restricted to patients with a history of mood disorders	8,638	6/4,012.2	1.50	23,503	6/11,876.5	0.51
Restricted to patients with a history of anxiety disorders	10,371	5/4,813.7	1.04	29,099	5/14,890.8	0.34
Restricted to patients with a history of other psychiatric disorder	10,413	4/4,910.2	0.81	27,989	7/13,989.4	0.50
Restricted to patients with a history of mood disorders, censoring at 90 days	8,638	3/1,473.6	2.04	23,503	4/4,296.0	0.93
Outcome: self-harm hospitalisation with admission to an intensive care unit						
Whole study population	69,786	13/31,344.9	0.41	217,577	16/110,329.4	0.15
Whole study population, censoring at 90 days maximum	69,786	8/11,689.5	0.68	217,577	6/39,082.2	0.15
Excluding patients with a history of psychiatric disorder or self-harm	49,962	4/22,013.3	0.18	161,993	7/82,073.9	0.09
Restricted to patients with a history of psychiatric disorder or self-harm	19,824	9/9,331.6	0.96	55,584	9/28,255.5	0.32
Restricted to patients with a history of self-harm within 3 years	185	3/79.8	37.58	466	3/209.4	14.33
Restricted to patients with a history of mood disorders	8,638	7/4,012.2	1.74	23,503	5/11,876.5	0.42
Restricted to patients with a history of anxiety disorders	10,371	7/4,813.7	1.45	29,099	4/14,890.8	0.27
Restricted to patients with a history of other psychiatric disorder	10,413	5/4,910.2	1.02	27,989	8/13,989.4	0.57
Restricted to patients with a history of mood disorders, censoring at 90 days	8,638	4/1,473.6	2.71	23,503	1/4,296.0	0.23

*Incidence rates are expressed per 1,000 person-years

**Self-harm with violent means defined by an ICD-10 code (X66-X83)

Appendix 6. Crude associations with suicide death or self-harm hospitalisation (composite outcome) for variables included in the IPTW treatment model

	HR	[95% CI]	p value
**Age***			
50-59 years	1.00		
60-69 years	0.41	[0.24;0.70]	0.001
70-79 years	0.77	[0.47;1.24]	0.277
80-89 years	1.10	[0.68;1.79]	0.700
90 years and over	1.77	[0.88;3.56]	0.108
**Year of treatment initiation**			
2012	1.00		
2013	1.05	[0.70;1.58]	0.816
2014	0.91	[0.59;1.41]	0.688
2015	1.29	[0.85;1.95]	0.236
2016 (January-June)	0.71	[0.37;1.39]	0.321
**Pre-existing psychiatric conditions and treatments**			
Self-harm hospitalisation	58.5	[36.8;93.0]	<0.001
Mood disorder or treatment for mood disorder	4.89	[3.63;6.57]	<0.001
Anxiety disorder or treatment for anxiety disorder	4.42	[3.30;5.92]	<0.001
Other psychiatric diagnoses or psychotropic treatments	4.92	[3.68;6.58]	<0.001
**Pre-existing prostatic conditions and treatments**			
Alpha-blockers	0.91	[0.68;1.22]	0.548
Transurethral resection of the prostate	0.75	[0.10;5.34]	0.773
Prostate cancer	0.96	[0.31;3.01]	0.945
**Pre-existing conditions used in the Charlson index**			
Myocardial infarct	0.95	[0.61;1.47]	0.824
Congestive heart failure	2.58	[1.69;3.93]	<0.001
Peripheral vascular disease	1.39	[0.91;2.12]	0.125
Cerebrovascular disease	1.58	[0.99;2.52]	0.053
Dementia	1.92	[1.07;3.45]	0.029
Chronic pulmonary disease	1.45	[1.05;2.01]	0.026
Connective tissue disease	1.45	[0.46;4.55]	0.521
Ulcer disease	1.30	[0.32;5.23]	0.713
Moderate or severe renal disease	2.28	[1.36;3.80]	0.002
Hemiplegia	1.82	[0.90;3.70]	0.098
HIV/AIDS	0.00	[0.00;Inf]	0.991
Liver disease	3.29	[1.68;6.43]	<0.001
Diabetes			
Diabetes without end-organ damage	0.71	[0.45;1.10]	0.126
Diabetes with end-organ damage	1.46	[0.79;2.69]	0.226
Cancer (except non melanoma skin cancer)			
Non metastatic cancer (including lymphoma and leukemia)	1.60	[1.09;2.36]	0.017
Metastatic solid tumor	2.67	[0.99;7.22]	0.053

Unadjusted Cox proportional hazards models conducted on the whole study population.
* Age was included as a quadratic polynomial in the IPTW treatment model
CI: confidence interval
HIV/AIDS: human immunodeficiency virus infection; acquired immunodeficiency syndrome
HR: hazard ratio
IPTW: inverse probability of treatment weighting

Appendix 7. Risk of suicide death or self-harm hospitalisation associated with finasteride relative to dutasteride exposure (unadjusted Cox proportional hazards models)

	HR	[95% CI]	p value
Outcome: suicide death or self-harm hospitalisation			
Whole study population	1.36	[0.99;1.88]	0.060
Whole study population, censoring at 90 days maximum	1.67	[1.09;2.55]	0.019
Excluding patients with a history of psychiatric disorder or self-harm	0.89	[0.47;1.68]	0.722
Restricted to patients with a history of psychiatric disorder or self-harm	1.44	[0.99;2.10]	0.057
Restricted to patients with a history of self-harm within 3 years	1.16	[0.44;3.01]	0.767
Restricted to patients with a history of mood disorders	1.61	[0.99;2.61]	0.057
Restricted to patients with a history of anxiety disorders	1.56	[0.97;2.50]	0.066
Restricted to patients with a history of other psychiatric disorder	1.30	[0.81;2.08]	0.282
Restricted to patients with a history of mood disorders, censoring at 90 days	1.70	[0.88;3.28]	0.117
Outcome: suicide death			
Whole study population	1.33	[0.77;2.28]	0.309
Whole study population, censoring at 90 days maximum	1.81	[0.89;3.65]	0.099
Excluding patients with a history of psychiatric disorder or self-harm	0.86	[0.33;2.29]	0.768
Restricted to patients with a history of psychiatric disorder or self-harm	1.51	[0.78;2.94]	0.226
Restricted to patients with a history of self-harm within 3 years	NE		
Restricted to patients with a history of mood disorders	2.35	[0.93;5.95]	0.072
Restricted to patients with a history of anxiety disorders	1.75	[0.73;4.16]	0.209
Restricted to patients with a history of other psychiatric disorder	1.71	[0.75;3.90]	0.205
Restricted to patients with a history of mood disorders, censoring at 90 days	4.70	[1.12;19.7]	0.034
Outcome: self-harm hospitalisation			
Whole study population	1.36	[0.92;2.03]	0.126
Whole study population, censoring at 90 days maximum	1.55	[0.91;2.64]	0.105
Excluding patients with a history of psychiatric disorder or self-harm	0.91	[0.40;2.09]	0.830
Restricted to patients with a history of psychiatric disorder or self-harm	1.38	[0.88;2.18]	0.162
Restricted to patients with a history of self-harm within 3 years	1.24	[0.47;3.26]	0.666
Restricted to patients with a history of mood disorders	1.40	[0.78;2.49]	0.256
Restricted to patients with a history of anxiety disorders	1.44	[0.82;2.53]	0.200
Restricted to patients with a history of other psychiatric disorder	1.14	[0.64;2.04]	0.655
Restricted to patients with a history of mood disorders, censoring at 90 days	1.26	[0.57;2.74]	0.569
Outcome: self-harm hospitalisation with violent means*			
Whole study population	1.85	[0.89;3.84]	0.098
Whole study population, censoring at 90 days maximum	2.08	[0.68;6.36]	0.199
Excluding patients with a history of psychiatric disorder or self-harm	1.02	[0.28;3.65]	0.980
Restricted to patients with a history of psychiatric disorder or self-harm	2.43	[0.96;6.16]	0.061
Restricted to patients with a history of self-harm within 3 years	1.28	[0.12;14.1]	0.842
Restricted to patients with a history of mood disorders	2.95	[0.95;9.17]	0.061
Restricted to patients with a history of anxiety disorders	3.09	[0.89;10.7]	0.075
Restricted to patients with a history of other psychiatric disorder	1.66	[0.49;5.68]	0.417
Restricted to patients with a history of mood disorders, censoring at 90 days	2.23	[0.50;9.98]	0.293
Outcome: self-harm hospitalisation with admission to an intensive care unit			
Whole study population	2.83	[1.36;5.88]	0.005
Whole study population, censoring at 90 days maximum	4.38	[1.52;12.6]	0.006
Excluding patients with a history of psychiatric disorder or self-harm	2.09	[0.61;7.15]	0.240
Restricted to patients with a history of psychiatric disorder or self-harm	3.01	[1.19;7.58]	0.020
Restricted to patients with a history of self-harm within 3 years	2.59	[0.52;12.8]	0.244
Restricted to patients with a history of mood disorders	4.06	[1.29;12.8]	0.017
Restricted to patients with a history of anxiety disorders	5.22	[1.53;17.9]	0.008
Restricted to patients with a history of other psychiatric disorder	1.80	[0.59;5.50]	0.304
Restricted to patients with a history of mood disorders, censoring at 90 days	11.4	[1.27;101.8]	0.030

*Self-harm with a violent mean defined by an ICD-10 code (X66-X83)

CI: confidence interval

HR: hazard ratio

NE: not estimable

Appendix 8. Propensity score stabilized weight distribution in IPTW adjusted analyses

Studied population	Stabilized weights distribution
Whole study population	mean=1.000; Q1-Q3=[0.930;1.055]; range=(0.400;2.279)
Whole study population, censoring at 90 days maximum	mean=1.000; Q1-Q3=[0.930;1.055]; range=(0.400;2.279)
Excluding patients with a history of psychiatric disorder or self-harm	mean=1.000; Q1-Q3=[0.934;1.051]; range=(0.394;2.330)
Restricted to patients with a history of psychiatric disorder or self-harm	mean=1.000; Q1-Q3=[0.921;1.061]; range=(0.447;2.177)
Restricted to patients with a history of self-harm within 3 years	mean=1.000; Q1-Q3=[0.856;1.060]; range=(0.338;4.809)
Restricted to patients with a history of mood disorders	mean=1.000; Q1-Q3=[0.922;1.066]; range=(0.441;1.999)
Restricted to patients with a history of anxiety disorders	mean=1.000; Q1-Q3=[0.925;1.061]; range=(0.412;2.192)
Restricted to patients with a history of other psychiatric disorder	mean=1.000; Q1-Q3=[0.915;1.066]; range=(0.460;2.246)
Restricted to patients with a history of mood disorders, censoring at 90 days	mean=1.000; Q1-Q3=[0.922;1.066]; range=(0.441;1.999)

IPTW: inverse probability of treatment weighting
Q1-Q3: first and fourth quartiles

Standardized differences before and after IPTW (analysis conducted on the whole study population)

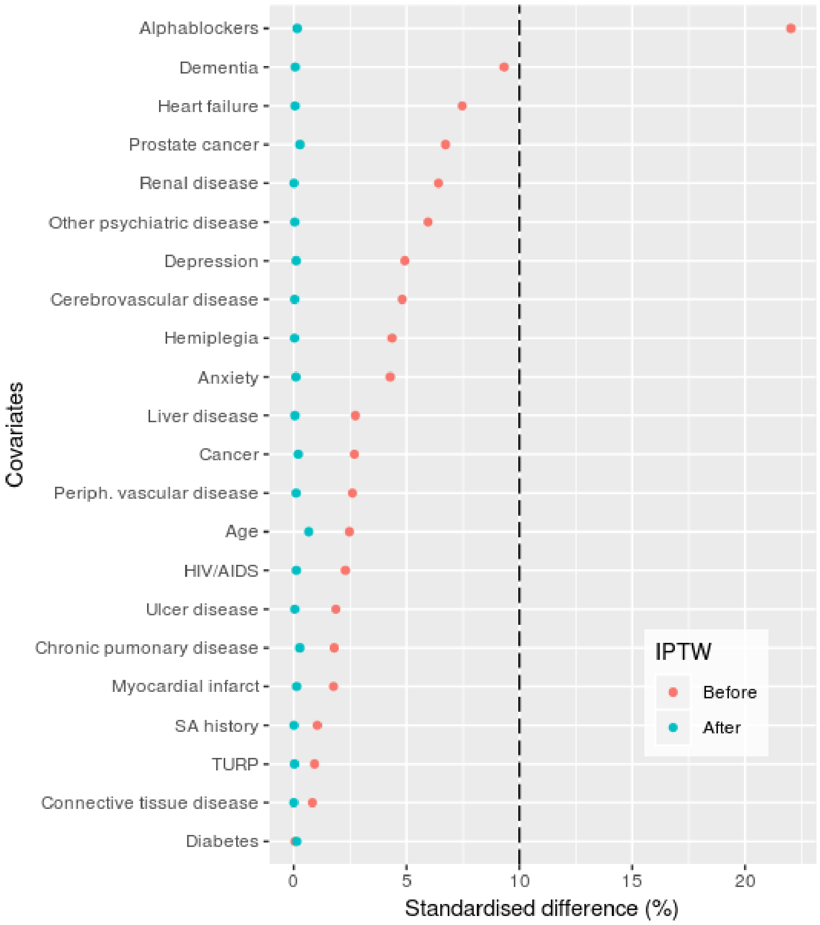

Age was adjusted for as a quadratic polynomial in the IPTW treatment model.
HIV/AIDS: human immunodeficiency virus infection; acquired immunodeficiency syndrome
IPTW: inverse probability of treatment weighting
TURP: transurethral resection of the prostate

Appendix 9. Risk of suicide death or self-harm hospitalisation associated with finasteride relative to dutasteride exposure (IPTW adjusted analyses, adjusting for age as a time-varying covariate)

	HR	[95% CI]	p value
Outcome: suicide death or self-harm hospitalisation			
Whole study population	1.11	[0.84;1.46]	0.463
Whole study population, censoring at 90 days maximum	1.54	[1.02;2.32]	0.041
Excluding patients with a history of psychiatric disorder or self-harm	0.56	[0.32;1.00]	0.050
Restricted to patients with a history of psychiatric disorder or self-harm	1.41	[1.02;1.94]	0.038
Restricted to patients with a history of self-harm within 3 years	0.89	[0.34;2.30]	0.811
Restricted to patients with a history of mood disorders	1.87	[1.25;2.80]	0.002
Restricted to patients with a history of anxiety disorders	1.52	[1.01;2.31]	0.047
Restricted to patients with a history of other psychiatric disorder	1.20	[0.80;1.81]	0.384
Restricted to patients with a history of mood disorders, censoring at 90 days	1.97	[1.07;3.64]	0.029
Outcome: suicide death			
Whole study population	1.15	[0.73;1.83]	0.549
Whole study population, censoring at 90 days maximum	1.64	[0.83;3.26]	0.156
Excluding patients with a history of psychiatric disorder or self-harm	0.64	[0.29;1.45]	0.288
Restricted to patients with a history of psychiatric disorder or self-harm	1.58	[0.89;2.80]	0.119
Restricted to patients with a history of self-harm within 3 years	NE		
Restricted to patients with a history of mood disorders	2.82	[1.30;6.10]	0.008
Restricted to patients with a history of anxiety disorders	1.79	[0.84;3.78]	0.130
Restricted to patients with a history of other psychiatric disorder	1.78	[0.84;3.78]	0.130
Restricted to patients with a history of mood disorders, censoring at 90 days	5.80	[1.43;23.5]	0.014
Outcome: self-harm hospitalisation			
Whole study population	1.07	[0.76;1.51]	0.688
Whole study population, censoring at 90 days maximum	1.45	[0.86;2.42]	0.160
Excluding patients with a history of psychiatric disorder or self-harm	0.50	[0.22;1.12]	0.091
Restricted to patients with a history of psychiatric disorder or self-harm	1.31	[0.89;1.93]	0.170
Restricted to patients with a history of self-harm within 3 years	0.99	[0.38;2.58]	0.983
Restricted to patients with a history of mood disorders	1.57	[0.98;2.53]	0.063
Restricted to patients with a history of anxiety disorders	1.38	[0.84;2.27]	0.206
Restricted to patients with a history of other psychiatric disorder	1.02	[0.62;1.67]	0.943
Restricted to patients with a history of mood disorders, censoring at 90 days	1.44	[0.70;2.95]	0.326
Outcome: self-harm hospitalisation with violent means*			
Whole study population	1.63	[0.89;2.98]	0.110
Whole study population, censoring at 90 days maximum	2.41	[0.83;6.94]	0.104
Excluding patients with a history of psychiatric disorder or self-harm	0.55	[0.16;1.88]	0.342
Restricted to patients with a history of psychiatric disorder or self-harm	2.94	[1.37;6.32]	0.006
Restricted to patients with a history of self-harm within 3 years	0.64	[0.07;6.13]	0.701
Restricted to patients with a history of mood disorders	4.74	[1.85;12.1]	0.001
Restricted to patients with a history of anxiety disorders	3.00	[0.95;9.47]	0.061
Restricted to patients with a history of other psychiatric disorder	1.37	[0.42;4.43]	0.604
Restricted to patients with a history of mood disorders, censoring at 90 days	3.00	[0.76;11.84]	0.117
Outcome: self-harm hospitalisation with admission to an intensive care unit			
Whole study population	2.20	[1.19;4.05]	0.012
Whole study population, censoring at 90 days maximum	3.74	[1.36;10.3]	0.011
Excluding patients with a history of psychiatric disorder or self-harm	1.11	[0.36;3.41]	0.858
Restricted to patients with a history of psychiatric disorder or self-harm	3.10	[1.44;6.68]	0.004
Restricted to patients with a history of self-harm within 3 years	2.01	[0.49;8.19]	0.331
Restricted to patients with a history of mood disorders	3.27	[1.34;7.99]	0.009
Restricted to patients with a history of anxiety disorders	4.66	[1.79;12.1]	0.002
Restricted to patients with a history of other psychiatric disorder	2.14	[0.91;5.07]	0.083
Restricted to patients with a history of mood disorders, censoring at 90 days	11.5	[1.31;101.1]	0.028

Cox proportional hazard models controlled by IPTW for the following potential confounders: age, year of treatment initiation, pre-existing prostatic and psychiatric conditions and treatments, comorbidities used in the Charlson index.

*Self-harm with violent means defined by an ICD-10 code (X66-X83)

CI: confidence interval

HR: hazard ratio

NE: not estimable
